# Differences in histomorphology and expression of key lipid regulated genes of four adipose tissues from Tibetan pigs

**DOI:** 10.7717/peerj.14556

**Published:** 2023-01-09

**Authors:** Chenghong Lin, Zexia Dong, Jia Song, Sutian Wang, Ying Yang, Hua Li, Zheng Feng, Yangli Pei

**Affiliations:** 1 Guangdong Provincial Key Laboratory of Animal Molecular Design and Precise Breeding, Key Laboratory of Animal Molecular Design and Precise Breeding of Guangdong Higher Education Institutes, School of Life Science and Engineering, Foshan University, Foshan, China; 2 State Key Laboratory of Livestock and Poultry Breeding, Guangdong Key Laboratory of Animal Breeding and Nutrition, Institute of Animal Science, Guangdong Academy of Agricultural Sciences, Guangzhou, China

**Keywords:** Tibetan pig, Adipocyte, Subcutaneous white adipose tissue, Perirenal fat, Greater omentum fat, Inguinal fat, FABP, PPARγ, C/EBPβ

## Abstract

Tibetan pigs, an indigenous pig breed in China, have high overall fat deposition and flavorful and tasty meat. They are thus good models for studying adipogenesis. Few studies have been conducted focusing on expression of lipid regulated genes in different adipose tissues of Tibetan pigs. Therefore, we compared the difference of histomorphology and expression level of lipid regulated genes through qPCR and western blot in subcutaneous fat, perirenal fat, omental adipose tissue, and inguinal fat of Tibetan pigs. Our results showed that the area of subcutaneous adipocytes in Tibetan pigs was smaller, while the other three adipose tissues (perirenal fat, greater omentum fat, inguinal fat) had cell areas of similar size. The gene expression of *FABP4*, *FASN*, *FABP3*, and *ME1* in subcutaneous fat was significantly higher than that in perirenal fat. Furthermore, the protein expression of FABP4 was significantly lower in subcutaneous fat than in perirenal fat (*p* < 0.05), and the expression of FASN was higher in greater omentum fat than in subcutaneous fat (*p* = 0.084). The difference in adipocyte cell size and expression of lipid-regulated genes in adipose tissues from the various parts of the pig body is likely due to the different cellular lipid metabolic processes. Specially, FABP4 and FASN may be involved in the regulation of fat deposition in different adipose tissues of Tibetan pigs.

## Introduction

Tibetan pigs, also known as ginseng pigs, are characterized by good meat flavor, strong respiratory system and robust limbs. Tibetan pigs are particularly popular in high-end markets, where their prices are considerably higher than those of other commercial pork varieties(Ai et al., 2014; Chen et al., 2014; Fan et al., 2003; Huang et al., 2021). Depending on the distribution, fat tissue is classified as either subcutaneous adipose tissue (SAT) or visceral adipose tissue (VAT). Subcutaneous fat lies between the skin and muscle, and visceral fat is located in the region around the internal organs(Gil et al., 2011; Ibrahim, 2010). Higher fat percentage and average backfat thickness were observed in Tibetan pigs than in Yorkshire pigs, and the fat composition of the two breeds also differed significantly. The monounsaturated fatty acid, hexadecenoic acid, and octadecenoic acid levels in subcutaneous fat were significantly higher in the Tibetan than in the Yorkshire pigs (Shang et al., 2019). Monounsaturated fatty acids are beneficial to human health (Shang et al., 2019). Increased monounsaturated intake improves sperm quality and fertility and assists reproductive technology success in men (Alesi et al., 2022). Considering the beneficial fat composition of Tibetan pigs, studying the mechanism of fat synthesis in Tibetan pig adipose tissue is of great interest.

In previous studies, fat cells from different anatomical sites exhibited different morphologies and cytokine and adipokine expression patterns (Rittig et al., 2012). For example, visceral fat contains larger adipocytes than subcutaneous fat in Bama pigs (Zhang et al., 2022b), and there are significant effects of adipocyte area and volume between breed and adipose tissues (Kolouchová et al., 2016; Merlotti et al., 2017). Differences in the metabolism of adipocytes from subcutaneous and visceral fat have been demonstrated (Lafontan & Berlan, 2003). The lipid content in perirenal fat was higher than that in subcutaneous fat from four age groups (80, 120, 160, and 210 days) in female Pietrain × (Large White × Landrace) pigs (Gardan, Gondret & Louveau, 2006). Adipocytes constitute the adipose tissue depot, and depot differences in subcutaneous and visceral fat are related to lipid metabolism and endocrine function in Bama pigs through comparison of adipocyte area and functional enrichment analysis (Zhang et al., 2022b). Zhou et al. (2022) showed that SAT displayed active organic acid metabolism and energy mobilization in Duroc × Landrace × Yorkshire (DLY) pigs upon exposure to cold. In contrast to SAT, cold-induced transcriptional changes are far less extensive in VAT (Zhou et al., 2022). Previous studies have mostly focused on single nucleotide polymorphisms (SNPs), gene expression in Tibetan pigs (Dong et al., 2015), or on mining differentially expressed genes between Tibetan pigs and other pig breeds using sequencing technology. The expression of lipid genes in different adipose tissues has been studied in humans and mice (Nono Nankam et al., 2020), but no related experiments have been conducted in Tibetan pigs.

Fat deposition is an important factor affecting meat quality, subcutaneous fat affects pork carcass value, and intramuscular fat affects pork flavor (Alfaia et al., 2019). Excessive subcutaneous fat deposition can reduce the growth performance and meat production efficiency of pigs (Liu et al., 2018).

Few studies have focused on the expression of lipid-regulated genes in the different adipose tissues of Tibetan pigs. Therefore, we compared differences in adipocyte area, expression of lipid-regulated genes (*PPARγ*, *C/EBPβ*, *FABP3*, *FABP4*, *FABP5*, *ACACA*, *FASN*, *ELOVL6*, *LPL*, *ME1*, *DGAT2*, *and SCD*), and protein expression in subcutaneous fat, perirenal fat, greater omentum fat, and inguinal fat of Tibetan pigs.

## Materials and Methods

### Animals, diets and tissue collection

Subcutaneous fat, perirenal fat, greater omentum fat, and inguinal fat were collected from five 8-month-old Tibetan pigs (the pigs for slaughter had been fasting for 12 h). Feed ingredients and nutrients composition of the experimental diet were provided in Table S1. Subcutaneous fat is collected from the inner layer of subcutaneous fat on the back fat. The adipose tissue was snap frozen in liquid nitrogen, and finally stored at −80 °C for subsequent experiments.

### HE staining

Paraffin sections (5 μm-thick) were dewaxed and rehydrated through a graded series of ethanol solutions. Then the adipose sections were washed in water. Hydrated sections were stained with 5% hematoxylin solution for 5 min, and rinsed with tap water. Then the sections were treated with hematoxylin differentiation solution, and rinsed with tap water again. Next, adipose tissue sections were stained in 0.5% eosin solution for 5 min. Finally, the sections were observed using a LEICA DMi8 microscope. Total adipocyte size (μm^2^) was measured by ImageJ (https://imagej.net/software/fiji/). Adipocyte size was calculated as the ratio of total adipocyte size/adipocyte number (cell counts are shown in Table S2), and incomplete cells were removed manually.

### Quantitative real-time reverse-transcription polymerase chain reaction (qRT-PCR)

Total RNA was extracted from adipose tissues using TRIzol reagent (Vazyme, Nanjing, China). RNA (1 μg) was reverse transcribed into cDNA using PrimeScript RT reagent kit with gDNA Eraser (RR047A; Takara, Kusatsu, Japan). Then cDNAs were amplified with TaKaRa SYBR Premix EX Taq (RR420A; Takara, Kusatsu, Japan) on a QuantStudio5 Real-time PCR Instrument (Thermo Fisher Scientific, Waltham, MA, USA). Beta-2-Microglobulin (*B2m*) was used as a housekeeping gene, and the 2^−(ΔΔct)^ method was used to calculate relative gene expression. Primer sequences are shown in Table S3.

### Western blot

Total proteins were lysed in radioimmunoprecipitation assay (RIPA) lysis buffer containing phenylmethylsulfonyl fluoride (PMSF) (Beyotime, Nantong, China) and phosphatase inhibitors. SDS-PAGE was used to separate adipose tissue lysates on a gradient of 6% sodium dodecyl sulfate-polyacrylamide gels (SDS-PAGE) for FASN, 12% SDS-PAGE for FABP4 and followed by electrotransferring them to Immobilon-P membranes (Merck Millipore, Burlington, MA, USA). After blocking in 5% skimmed milk for 2 h (25 °C), membranes were probed as indicated with FASN (1:1,000, 10624-2-AP, Proteintech), FABP4 (1:1,000, 12802-1-AP, Proteintech), and α-Tublin (1:10,000, 11224-1-AP, Proteintech) antibodies for 2 h, then membranes were incubated with horseradish peroxidase (HRP)-labeled goat anti rabbit IgG antibody for 40 min. The indicated proteins were visualized using an enhanced chemiluminescence (ECL) reagent (Thermo Fisher Scientific, Waltham, MA, USA).

### Statistical analysis

Three independent experiments were conducted. Data were expressed as mean ± SEM and analyzed by SPSS 26.0 software using one-way ANOVA. *p* < 0.05 was considered to be significant (*p < 0.05).

## Results

### Adipocyte morphology, distribution, and size of four adipose tissues

**Figure 1:**
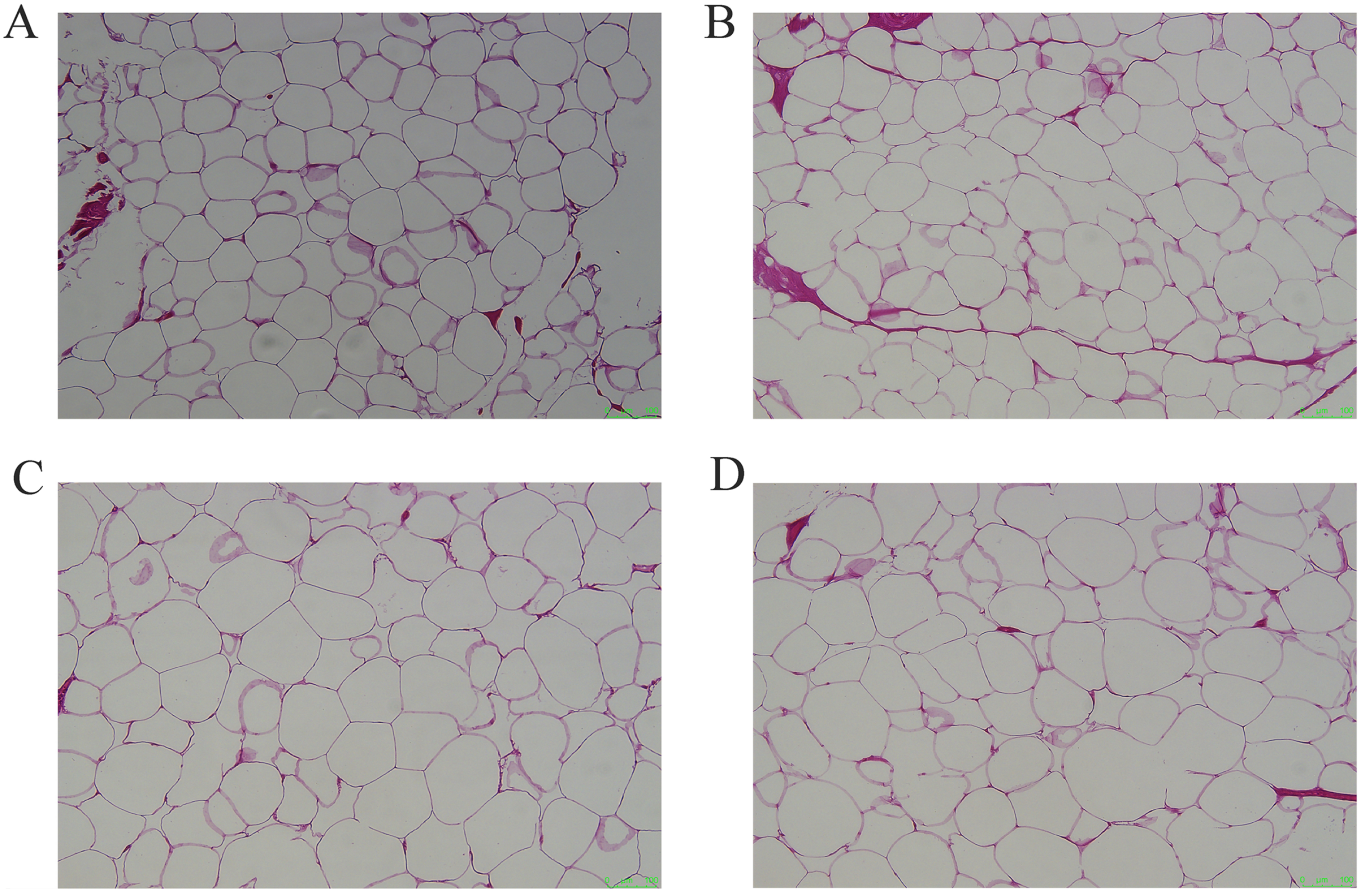
Representative pictures of HE staining of different adipose tissues. (A) HE staining of subcutaneous adipose tissue, (B) perirenal adipose tissue, (C) omental adipose tissue, (D) inguinal adipose tissue. Scale bars, 100 μm.

**Figure 2:**
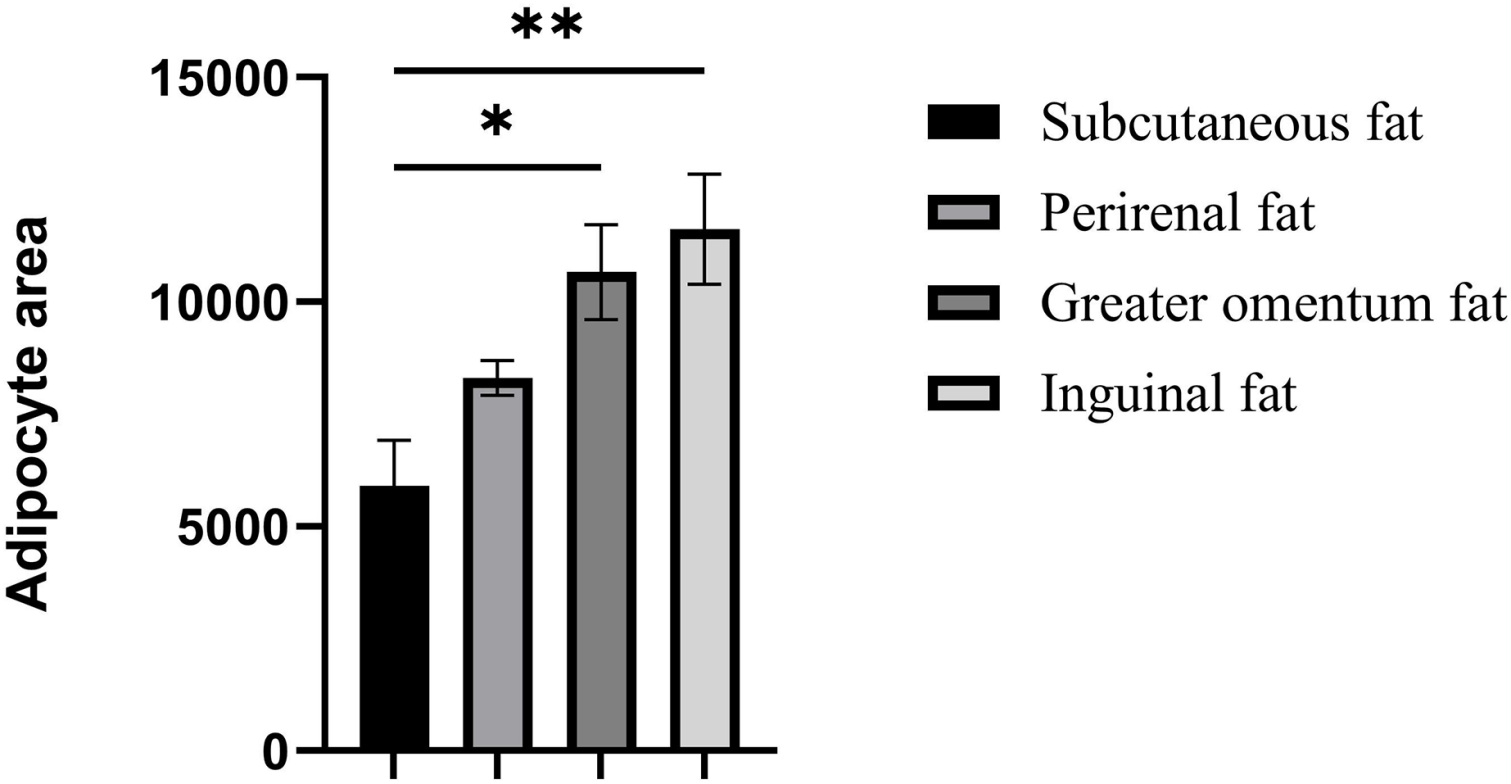
Assessment of adipocyte area in four adipose tissues. Assessment of adipocyte area in four adipose tissues. Each column represents the mean ± SEM (*n* = 7). *p < 0.05, **p < 0.01.

The cellular morphology of the four adipose tissues was round or oval with individual well-circumscribed cell borders (Fig. 1). The adipocyte area was measured using the ImageJ software. The adipocyte area of subcutaneous fat (5,906.78 ± 1,011.90 μm^2^) in Tibetan pigs was smaller than that of perirenal fat (8,306.20 ± 840 μm^2^), and greater omentum fat (10,665.87 ± 1,056.47 μm^2^) was similar to inguinal fat (11,614.53 ± 1,226.49 μm^2^). The adipocyte area of the greater omentum and inguinal fat was larger than that of subcutaneous fat (*p* < 0.05) (Fig. 2), and that of perirenal fat was not significantly different from that of other adipose tissues.

### mRNA expression of lipid-regulated genes

**Figure 3:**
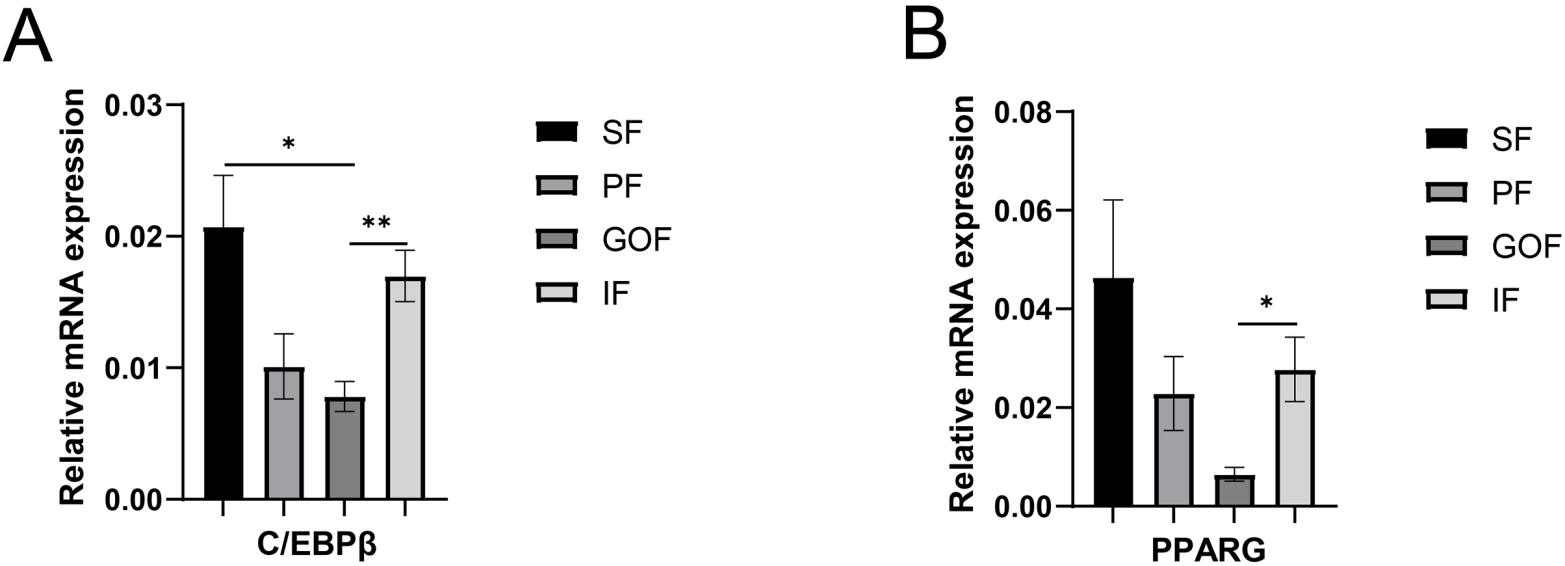
Validation of mRNA level of transcription factor genes. (A) Expression of *PPARγ*; *e*xpression of *C/EBPβ* (B). Each column represents mean ± SEM (*n* = 5). Subcutaneous fat (SF), perirenal fat (PF), greater omentum fat (GOF), inguinal fat (IF). *p < 0.05, **p < 0.01.

**Figure 4:**
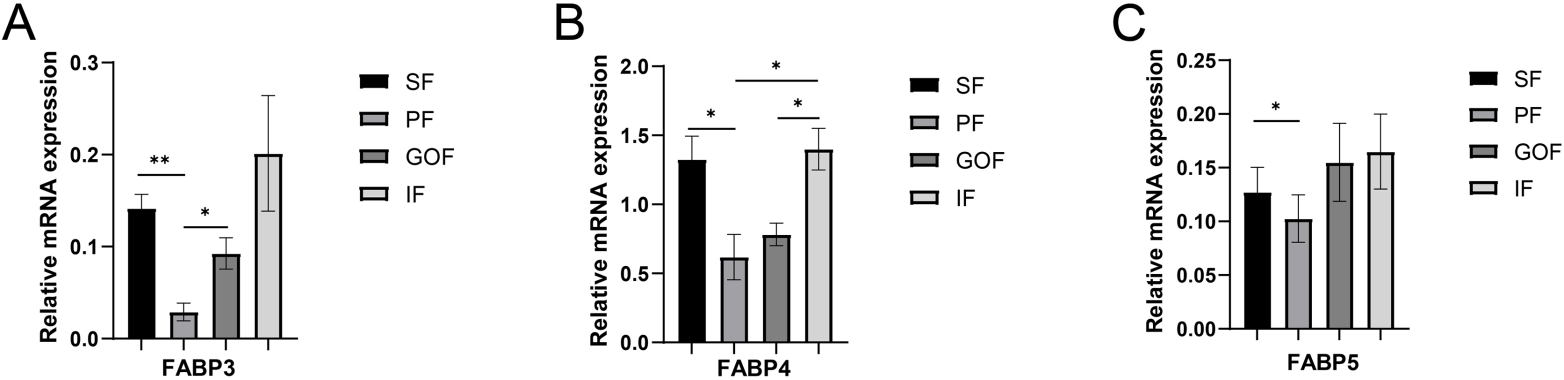
Validation of mRNA levels of fatty acid transportation genes. (A) Expression of FABP3; (B) expression of FABP4; (C) expression of FABP5. Each column represents mean ± SEM (*n* = 5). *p < 0.05, **p < 0.01.

**Figure 5:**
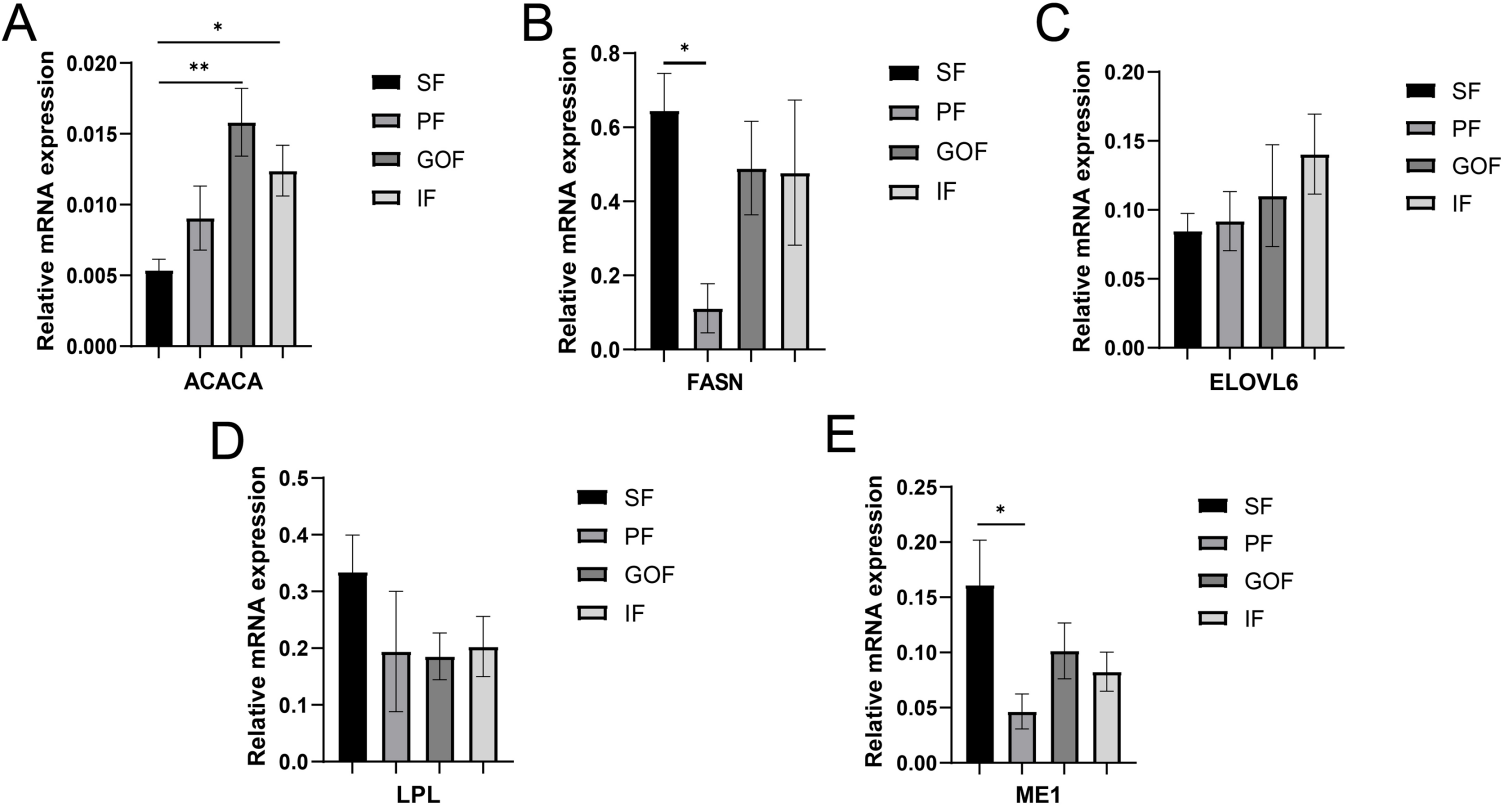
Validation of mRNA levels of lipogenesis genes. (A) Expression of ACACA; (B) expression of FASN; (C) expression of ELOVL6; (D) expression of LPL; expression of ME1. Each column represents mean ± SEM (*n* = 5). *p < 0.05, **p < 0.01.

**Figure 6:**
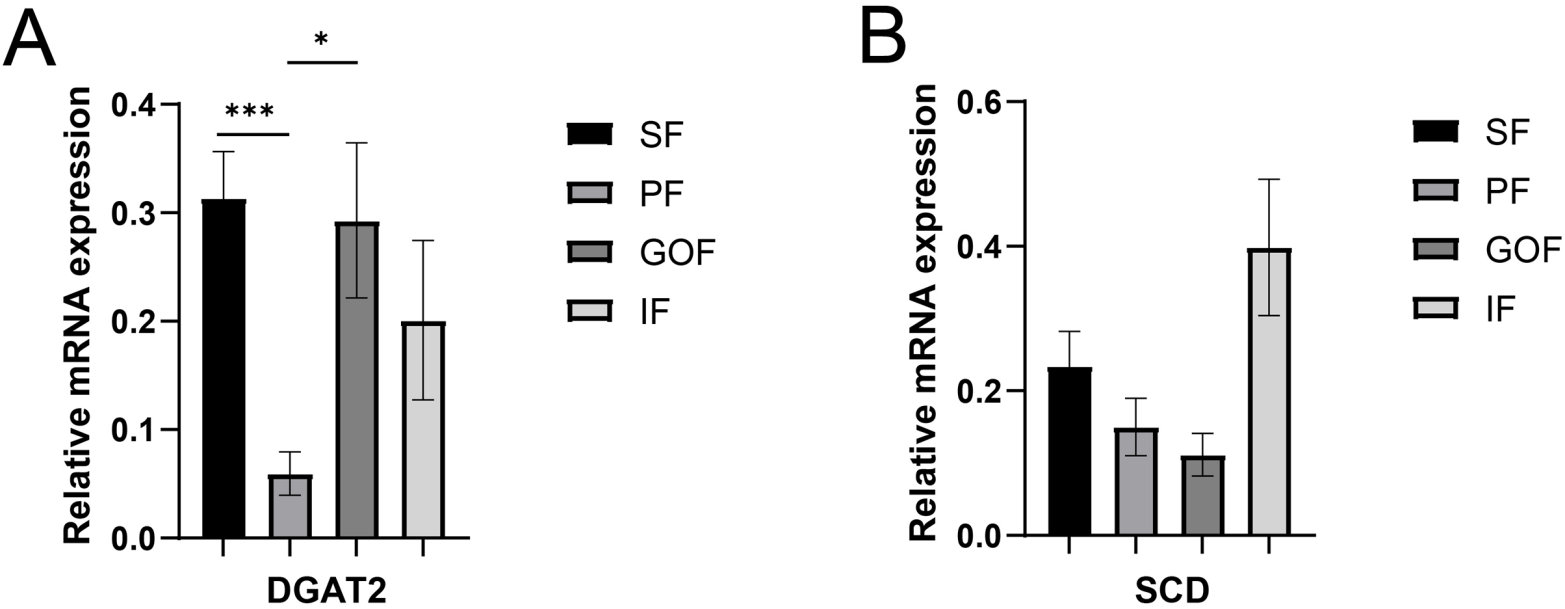
Validation of mRNA levels of fatty acid esterification genes. (A) Expression of DGAT2; (B) expression of SCD. Each column represents mean ± SEM (*n* = 5). *p < 0.05, ***p < 0.005.

Several lipid-related genes directly or indirectly affect fat deposition in pigs. Here, we detected mRNA expression levels of genes related to lipid metabolism. mRNA expression of Peroxisome proliferator-activated receptor γ (*PPARγ*) in greater omentum fat was lower than inguinal fat in a comparison of the four adipose tissues (*p* < 0.05) (Fig. 3A). The mRNA abundance of CCAAT/enhancer binding protein beta (*C/EBPβ*) was higher in subcutaneous fat (*p* < 0.05) and inguinal fat (*p* < 0.01) than in omentum fat (Fig. 3B). We also examined the expression levels of the three FABP family members in different adipose tissues. There were significant differences in the expression levels of fatty acid-binding protein 3 (*FABP3*) in subcutaneous fat, greater omentum fat, and perirenal fat (Fig. 4A). The mRNA expression levels of fatty acid-binding protein 4 (*FABP4*) in perirenal fat were significantly lower than those in subcutaneous fat and inguinal fat (*p* < 0.05), besides, expression of FABP4 in inguinal fat was higher than that in greater omentum fat (*p* < 0.05, Fig. 4B). Fatty acid-binding protein 5 (*FABP5*) was highly expressed in subcutaneous fat compared to perirenal fat (*p* < 0.05, Fig. 4C). Subsequently, we examined the expression levels of lipogenic genes. The expression level of fatty acid synthase (*FASN*) was higher in subcutaneous fat than in perirenal fat (*p* < 0.05, Fig. 5B), and the expression of malic enzyme 1 (*ME1*) was similar to that of *FABP5* (*p* < 0.05, Fig. 5E). Acetyl-CoA carboxylase alpha (*ACACA*) had higher expression in greater omentum fat (*p* < 0.01) and inguinal fat (*p* < 0.05) than in subcutaneous fat (Fig. 5A). There were no significant differences in the expression of ELOVL fatty acid elongase 6 (*ELOVL6*), lipoprotein lipase (*LPL*), or stearoyl-CoA desaturase (*SCD*) (Figs. 5C, 5D, and 6B). The expression of diacylglycerol O-acyltransferase 2 (*DGAT2*) was higher in subcutaneous fat (*p* < 0.005) and omentum fat (*p* < 0.05) than in perirenal fat (Fig. 6A).

### Expression of FASN and FABP4 in four tissue types

**Figure 7:**
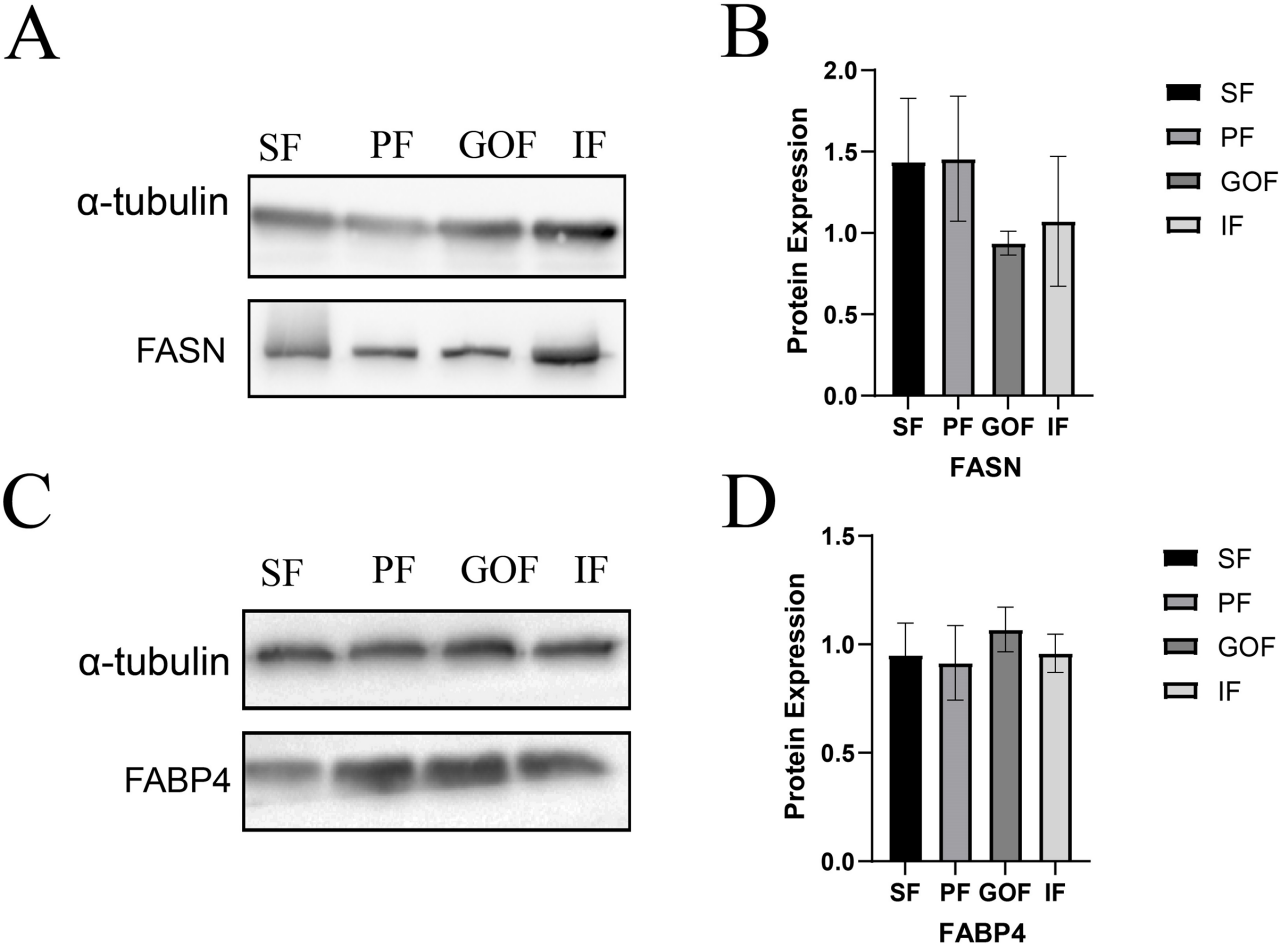
Expression of FASN and FABP4 proteins in four tissues. (A and B) Expression of FASN protein in four tissues; (C and D) Expression of FABP4 protein in four tissues. Each column represents the mean ± SEM (*n* = 5).

Subsequently, we detected the protein expression levels of FASN and FABP4 using western blots. The expression of FASN did not differ significantly among the four tissues (Fig. 7A). The expression level of FABP4 was similar in four adipose tissues. (Fig. 7B).

## Discussion

In the pig industry, low subcutaneous fat can improve carcass prices. Economically important pig breeds such as the Large White and Duroc have thin subcutaneous fat, while Tibetan pigs have high fat-deposition levels and delicious and nutritious meat (Gan et al., 2019; Shen et al., 2014). Differences in morphology and lipid-regulated genes also exist between breeds and adipose tissue types (Lopes et al., 2014).

The size of the lipid droplets determines the area of adipocytes to some extent, which can influence lipid deposition in animals or humans. In Meishan pigs, 80% of the subcutaneous fat mass was derived from 95–165 μm adipocytes at 5 months, whereas the subcutaneous fat of Landrace pigs was mainly 75–145 μm adipocytes at 5 months (Nakajima et al., 2011). In female Ossabaw swine (5–6 weeks old), adipocytes from the greater omentum fat were more than twice as large in the obese group than in the lean group (Toedebusch et al., 2014). The adipocyte area at 30 days of fattening in Iberian pigs was close to 13,693.49 ± 3,085.12 μm^2^ and 26,252.38 ± 8,429.56 μm^2^ at 90 days of fattening, with increases in the adipocyte area of backfat as the pigs gained weight. The adipocyte area was also positively correlated with the fattening time of pigs (Ayuso et al., 2018). In female Pietrain × (Large White × Landrace) pigs, the diameter of the subcutaneous adipocytes was 81 ± 4 μm at 210 days of age (Gardan, Gondret & Louveau, 2006). Consistent with the results of other pig breeds, the size of adipocytes differed between adipose tissue types of Tibetan pigs. Most of the adipocyte area is occupied by lipid droplets that are rich in triglycerides and cholesterol lipids. Many genes regulate lipid droplet size and deposition. Forty candidate genes that may regulate fat deposition in subcutaneous and intramuscular fat were identified using transcriptomics in Ding Yuan pigs (Zhang et al., 2022a). In addition, based on transcriptomes of different breeds (Landrace pigs, Tibetan pigs, and Rongchang pigs) and tissues (SAT and VAT), Zhou et al. (2013) suggested that subcutaneous adipose tissue differs from visceral and intermuscular adipose tissues due to lipid metabolism regulation (Zhou et al., 2013).

DGAT2, a member of the DGAT2/acyl-CoA: monoacylglycerol acyltransferase family, catalyzes triglyceride synthesis and promotes the formation of large lipid droplets (Cases et al., 2001). DGAT2 is highly expressed in adipose tissue, liver, small intestine, and mammary glands and has a robust capacity for triglyceride (TG) synthesis and storage (Yen et al., 2008), and is the major enzyme involved in TG synthesis in eukaryotes. When DGAT2 is overexpressed in cells in culture, mouse liver, or skeletal muscle, very high levels of TG are deposited in the cytosolic lipid droplets (Monetti et al., 2007; Stone et al., 2004). In contrast, almost no TG was present in mice lacking DGAT2 (Stone et al., 2004). A high-fat diet significantly increased the relative expression of DGAT2 in the backfat and liver tissues and induced obesity in genetically lean pigs (Yang et al., 2018). However, differences in adipose tissue between the different sites were not compared. Here, we found that the highest expression of *DGAT2* was in the subcutaneous fat, which had the smallest number of adipocytes. This may be due to subtle differences in the regulatory functions of DGAT2 in different species and tissues.

The expression of *PPARγ* in inguinal fat was higher than that in greater omentum fat. *C/EBPβ* and *PPARγ* showed similar expression trends in all four adipose tissues. Various transcription factors and adipose-secreting factors regulate fat deposition (Louveau et al., 2016). Transcriptional cascade regulation is the most important regulator of adipocyte differentiation and is strictly regulated by a series of transcription factors (C/EBP and PPARs). PPARγ also promotes preadipocytes maturation and as a transcription factor in the PPAR signaling pathway for downstream genes, including *LPL*, *FABP3*, and *ME1* (Achari & Jain, 2017). The mRNA and protein levels of PPAR*γ* were significantly higher in subcutaneous fat than in the intramuscular fat base in 7-months-old male Erhualian pigs (Wei et al., 2017). C/EBPβ is a transcription factor that inhibits preadipocyte adipogenesis in the subcutaneous fat (Yan et al., 2013). The gene expression of *PPARγ* and *C/EBPβ* was higher in subcutaneous fat than in other adipose tissues (PF, GOF, IF), suggesting that PPARγ and C/EBPβ participate in adipocyte differentiation, which may, in turn, affect the size of adipocytes in different adipose tissues.

ME1 converts malate to pyruvate while also generating NADPH from NADP in the cytosol (Merritt et al., 2009). Because it produces NADPH, a cofactor for fatty acid and cholesterol biosynthesis, and regulates reversible oxidative stress, ME1 plays an important role in lipid and cholesterol biosynthesis (Simmen, Alhallak & Simmen, 2020). Diets high in fat and carbohydrates cause high expression of the ME1 gene in rats, and the pattern of gene expression is consistent with increased lipid synthesis and storage (López et al., 2003). Previous studies have shown that *ME1* can affect the thickness of porcine dorsal muscles (Vidal et al., 2006), and our results showed that the mRNA expression level of *ME1* in subcutaneous fat was significantly higher than that in perirenal fat. Differences in the expression pattern of ME1 may be one reason for the different sizes of adipocytes in adipose tissues at different sites.

Three FABP isoforms, FABP3 (heart-type fatty acid-binding protein, H-FABP), FABP4 (adipocyte-type fatty acid-binding protein, A-FABP), and FABP5 (epidermal-type fatty acid-binding protein, E-FABP), have been identified in these adipose tissues (Hotamisligil & Bernlohr, 2015). FABPs directly function in the uptake, transport, and metabolic regulation of long-chain fatty acids (Nakamura, Yudell & Loor, 2014). FABP3 regulates solubility, mobility, and utilization of fatty acids as lipid chaperones (Furuhashi & Hotamisligil, 2008). FABP4 exists primarily in adipose tissue, which is vital for lipid metabolism in adipocytes (Falcao-Pires et al., 2012). FABP5, a lipid chaperone, regulates fatty acid transport, metabolism, and storage (Liu et al., 2018; Villaplana-Velasco et al., 2021; Xing et al., 2021). FABPs are candidate genes that regulate porcine fat deposition in many other studies. FABPs promote fatty acid solubilization, transport, and metabolism and interact with PPARs and hormone-sensitive lipase (HSL) to regulate cellular lipid responses (Li et al., 2020). The mRNA levels of *FABP3*, *FABP4*, and *FABP5* in subcutaneous and inguinal fat were significantly higher than those in perirenal fat. However, the protein expression levels of FABP4 showed no significant differences among the four adipose tissues. Therefore, the trends in FABP4 gene and protein expression were inconsistent. It is possible that *FABP4* has other regulatory functions at the translation level. Because of the highly regulated process of translating mRNAs into proteins, transcript levels do not always correlate with protein levels. Although the gene expression levels of *FABP4* did not correlate with protein expression, it was clear that the expression levels of *FABP3*, *FABP4*, and *FABP5* were significantly different among different adipose tissues at the transcriptional level. These results suggested that FABPs may have different biological functions in different adipose tissues.

FASN is a key rate-limiting enzyme in *de novo* lipogenesis that can rapidly synthesize fatty acids (Song, Xiaoli & Yang, 2018). FASN is associated with fat deposition in pigs and sheep (Xing et al., 2021; Zhang et al., 2019; Zhao et al., 2022). Zhang et al. (2022a) considered *FASN* a candidate gene for fat deposition. Another study showed a strong correlation between *FASN* and intramuscular fat (Wang et al., 2020), and FASN determined lipid deposition using transcriptome and proteome data (Wang et al., 2021). The mRNA expression of *FASN* in the subcutaneous fat was higher than that in the perirenal fat. A previous study showed that *FASN* and *SCD* levels were upregulated in intramuscular fat of the longissimus dorsi muscle in Diannan small-ear and Tibetan pigs compared to Landrace and Yorkshire pigs; thus, *FASN* and *SCD* were suggested to be key genes related to lipid deposition (Wang et al., 2015). Based on the results of this experiment, gene expression of FASN differed only between SF and PF, and no difference in the expression of FASN protein was observed between different tissues in Tibetan pigs. As a key gene in *de novo* lipogenesis, FASN converts malonyl-CoA to palmitate (Song, Xiaoli & Yang, 2018), and the function of FASN is likely to be the same across different adipose tissues.

## Conclusions

Adipocyte area of four adipose tissues (subcutaneous white adipose tissue; perirenal fat; greater omentum fat; inguinal fat) show significant differences in Tibetan pigs, this result may be caused by the regulation of lipid genes, such as *FABP3*, *FABP4*, *FABP5*, *C/EBPβ*, *PPARγ*, *DGAT2*, *ME1*, and *ACACA*. Lipid metabolism in different of adipose tissue in pigs has its unique molecular regulation mechanism. It can provide a basis for selection and breeding pigs with different fat characteristics at different parts of body.

## Supplemental Information

10.7717/peerj.14556/supp-1 Supplemental Information 1 Supplemental Tables.

10.7717/peerj.14556/supp-2 Supplemental Information 2 The original WB images, including the original image of Figure 7A and other WB images used in this article.

## Additional Information and Declarations

### Data Availability

The following information was supplied regarding data availability: The raw measurements are available in the Supplemental Files.

## References

[ref-1] Achari AE, Jain SK (2017). Adiponectin, a therapeutic target for obesity, diabetes, and endothelial dysfunction. International Journal of Molecular Sciences.

[ref-2] Ai H, Yang B, Li J, Xie X, Chen H, Ren J (2014). Population history and genomic signatures for high-altitude adaptation in Tibetan pigs. BMC Genomics.

[ref-3] Alesi S, Villani A, Mantzioris E, Takele WW, Cowan S, Moran LJ, Mousa A (2022). Anti-inflammatory diets in fertility: an evidence review. Nutrients.

[ref-4] Alfaia CM, Lopes PA, Madeira MS, Pestana JM, Coelho D, Toldra F, Prates JAM (2019). Current feeding strategies to improve pork intramuscular fat content and its nutritional quality. Advances in Food and Nutrition Research.

[ref-5] Ayuso D, Gonzalez A, Pena F, Izquierdo M (2018). Changes in adipose cells of Longissimus dorsi muscle in Iberian pigs raised under extensive conditions. Anais da Academia Brasileira de Ciências.

[ref-6] Cases S, Stone SJ, Zhou P, Yen E, Tow B, Lardizabal KD, Voelker T, Farese RV (2001). Cloning of DGAT2, a second mammalian diacylglycerol acyltransferase, and related family members. Journal of Biological Chemistry.

[ref-7] Chen A, Hao LL, Fang XB, Lu K, Liu SC, Zhang YL (2014). Polymorphism analysis of IGFBP-5 gene exon 1 in Tibet Mini-pig and Junmu No. 1 White pig. Genetics and Molecular Research.

[ref-8] Dong K, Pu Y, Yao N, Shu G, Liu X, He X, Zhao Q, Guan W, Ma Y (2015). Copy number variation detection using SNP genotyping arrays in three Chinese pig breeds. Animal Genetics.

[ref-9] Falcao-Pires I, Castro-Chaves P, Miranda-Silva D, Lourenco AP, Leite-Moreira AF (2012). Physiological, pathological and potential therapeutic roles of adipokines. Drug Discovery Today.

[ref-10] Fan B, Yang SL, Liu B, Yu M, Zhao SH, Li K (2003). Characterization of the genetic diversity on natural populations of Chinese miniature pig breeds. Animal Genetics.

[ref-11] Furuhashi M, Hotamisligil GS (2008). Fatty acid-binding proteins: role in metabolic diseases and potential as drug targets. Nature Reviews Drug Discovery.

[ref-12] Gan M, Shen L, Fan Y, Guo Z, Liu B, Chen L, Tang G, Jiang Y, Li X, Zhang S, Bai L, Zhu L (2019). High altitude adaptability and meat quality in tibetan pigs: a reference for local pork processing and genetic improvement. Animals.

[ref-13] Gardan D, Gondret F, Louveau I (2006). Lipid metabolism and secretory function of porcine intramuscular adipocytes compared with subcutaneous and perirenal adipocytes. American Journal of Physiology-Endocrinology and Metabolism.

[ref-14] Gil A, Olza J, Gil-Campos M, Gomez-Llorente C, Aguilera CM (2011). Is adipose tissue metabolically different at different sites?. International Journal of Pediatric Obesity.

[ref-15] Hotamisligil GS, Bernlohr DA (2015). Metabolic functions of FABPs—mechanisms and therapeutic implications. Nature Reviews Endocrinology.

[ref-16] Huang Z, Li Q, Li M, Li C (2021). Transcriptome analysis reveals the long intergenic noncoding RNAs contributed to skeletal muscle differences between Yorkshire and Tibetan pig. Scientific Reports.

[ref-17] Ibrahim MM (2010). Subcutaneous and visceral adipose tissue: structural and functional differences. Obesity Reviews.

[ref-18] Kolouchová I, Maťátková O, Sigler K, Masák J, Řezanka T (2016). Production of palmitoleic and linoleic acid in oleaginous and nonoleaginous yeast biomass. International Journal of Analytical Chemistry.

[ref-19] Lafontan M, Berlan M (2003). Do regional differences in adipocyte biology provide new pathophysiological insights?. Trends in Pharmacological Sciences.

[ref-20] Li B, Hao J, Zeng J, Sauter ER (2020). SnapShot: FABP functions. Cell.

[ref-21] Liu X, Liu K, Shan B, Wei S, Li D, Han H, Wei W, Chen J, Liu H, Zhang L (2018). A genome-wide landscape of mRNAs, lncRNAs, and circRNAs during subcutaneous adipogenesis in pigs. Journal of Animal Science and Biotechnology.

[ref-22] Lopes PA, Costa AS, Costa P, Pires VM, Madeira MS, Achega F, Pinto RM, Prates JA (2014). Contrasting cellularity on fat deposition in the subcutaneous adipose tissue and longissimus lumborum muscle from lean and fat pigs under dietary protein reduction. Animal.

[ref-23] Louveau I, Perruchot M-H, Bonnet M, Gondret F (2016). Invited review: pre- and postnatal adipose tissue development in farm animals: from stem cells to adipocyte physiology. Animal.

[ref-24] López IP, Marti A, Milagro FI, de los Angeles Zulet Md M, Moreno-Aliaga MJ, Martinez JA, De Miguel C (2003). DNA microarray analysis of genes differentially expressed in diet-induced (cafeteria) obese rats. Obesity Research.

[ref-25] Merlotti C, Ceriani V, Morabito A, Pontiroli AE (2017). Subcutaneous fat loss is greater than visceral fat loss with diet and exercise, weight-loss promoting drugs and bariatric surgery: a critical review and meta-analysis. International Journal of Obesity.

[ref-26] Merritt TJS, Kuczynski C, Sezgin E, Zhu C-T, Kumagai S, Eanes WF (2009). Quantifying interactions within the NADP(H) enzyme network in *Drosophila melanogaster*. Genetics.

[ref-27] Monetti M, Levin MC, Watt MJ, Sajan MP, Marmor S, Hubbard BK, Stevens RD, Bain JR, Newgard CB, Farese RV, Hevener AL, Farese RV (2007). Dissociation of hepatic steatosis and insulin resistance in mice overexpressing DGAT in the liver. Cell Metabolism.

[ref-28] Nakajima I, Oe M, Ojima K, Muroya S, Shibata M, Chikuni K (2011). Cellularity of developing subcutaneous adipose tissue in Landrace and Meishan pigs: adipocyte size differences between two breeds. Animal Science Journal.

[ref-29] Nakamura MT, Yudell BE, Loor JJ (2014). Regulation of energy metabolism by long-chain fatty acids. Progress in Lipid Research.

[ref-30] Nono Nankam PA, Bluher M, Kehr S, Kloting N, Krohn K, Adams K, Stadler PF, Mendham AE, Goedecke JH (2020). Distinct abdominal and gluteal adipose tissue transcriptome signatures are altered by exercise training in African women with obesity. Scientific Reports.

[ref-31] Rittig K, Dolderer JH, Balletshofer B, Machann J, Schick F, Meile T, Küper M, Stock UA, Staiger H, Machicao F, Schaller H-E, Königsrainer A, Häring H-U, Siegel-Axel DI (2012). The secretion pattern of perivascular fat cells is different from that of subcutaneous and visceral fat cells. Diabetologia.

[ref-32] Shang P, Li W, Liu G, Zhang J, Li M, Wu L, Wang K, Chamba Y (2019). Identification of lncRNAs and genes responsible for fatness and fatty acid composition traits between the tibetan and yorkshire pigs. International Journal of Genomics.

[ref-33] Shen L, Lei H, Zhang S, Li X, Li M, Jiang X, Zhu K, Zhu L (2014). The comparison of energy metabolism and meat quality among three pig breeds. Animal Science Journal.

[ref-34] Simmen FA, Alhallak I, Simmen RCM (2020). Malic enzyme 1 (ME1) in the biology of cancer: it is not just intermediary metabolism. Journal of Molecular Endocrinology.

[ref-35] Song Z, Xiaoli AM, Yang F (2018). Regulation and metabolic significance of de novo lipogenesis in adipose tissues. Nutrients.

[ref-36] Stone SJ, Myers HM, Watkins SM, Brown BE, Feingold KR, Elias PM, Farese RV (2004). Lipopenia and skin barrier abnormalities in DGAT2-deficient mice. Journal of Biological Chemistry.

[ref-37] Toedebusch RG, Roberts MD, Wells KD, Company JM, Kanosky KM, Padilla J, Jenkins NT, Perfield JW, Ibdah JA, Booth FW, Rector RS (2014). Unique transcriptomic signature of omental adipose tissue in Ossabaw swine: a model of childhood obesity. Physiological Genomics.

[ref-38] Vidal O, Varona L, Oliver MA, Noguera JL, Sanchez A, Amills M (2006). Malic enzyme 1 genotype is associated with backfat thickness and meat quality traits in pigs. Animal Genetics.

[ref-39] Villaplana-Velasco A, Noguera JL, Pena RN, Ballester M, Muñoz L, González E, Tejeda JF, Ibáñez-Escriche N (2021). Comparative transcriptome profile between iberian pig varieties provides new insights into their distinct fat deposition and fatty acids content. Animals.

[ref-40] Wang Z, Li Q, Chamba Y, Zhang B, Shang P, Zhang H, Wu C (2015). Identification of genes related to growth and lipid deposition from transcriptome profiles of pig muscle tissue. PLOS ONE.

[ref-41] Wang H, Wang J, Yang D-D, Liu Z-L, Zeng Y-Q, Chen W (2020). Expression of lipid metabolism genes provides new insights into intramuscular fat deposition in Laiwu pigs. Asian-Australasian Journal of Animal Sciences.

[ref-42] Wang L, Zhang Y, Zhang B, Zhong H, Lu Y, Zhang H (2021). Candidate gene screening for lipid deposition using combined transcriptomic and proteomic data from Nanyang black pigs. BMC Genomics.

[ref-43] Wei W, Sun W, Han H, Chu W, Zhang L, Chen J (2017). miR-130a regulates differential lipid accumulation between intramuscular and subcutaneous adipose tissues of pigs via suppressing PPARG expression. Gene.

[ref-44] Xing K, Liu H, Zhang F, Liu Y, Shi Y, Ding X, Wang C (2021). Identification of key genes affecting porcine fat deposition based on co-expression network analysis of weighted genes. Journal of Animal Science and Biotechnology.

[ref-45] Yan X, Weijun P, Ning W, Yu W, Wenkai R, Gongshe Y (2013). Knockdown of both FoxO1 and C/EBPβ promotes adipogenesis in porcine preadipocytes through feedback regulation. Cell Biology International.

[ref-46] Yang X-F, Qiu Y-Q, Wang L, Gao K-G, Jiang Z-Y (2018). A high-fat diet increases body fat mass and up-regulates expression of genes related to adipogenesis and inflammation in a genetically lean pig. Journal of Zhejiang University-Science B.

[ref-47] Yen CL, Stone SJ, Koliwad S, Harris C, Farese RV (2008). Thematic review series: glycerolipids. DGAT enzymes and triacylglycerol biosynthesis. Journal of Lipid Research.

[ref-48] Zhang P, Li Q, Wu Y, Zhang Y, Zhang B, Zhang H (2022a). Identification of candidate genes that specifically regulate subcutaneous and intramuscular fat deposition using transcriptomic and proteomic profiles in Dingyuan pigs. Scientific Reports.

[ref-49] Zhang Y, Wang H, Tu W, Abbas Raza SH, Cao J, Huang J, Wu H, Fan C, Wang S, Zhao Y, Tan Y (2022b). Comparative transcriptome analysis provides insight into spatio-temporal expression characteristics and genetic regulatory network in postnatal developing subcutaneous and visceral fat of bama pig. Frontiers in Genetics.

[ref-50] Zhang Y, Zhang J, Gong H, Cui L, Zhang W, Ma J, Chen C, Ai H, Xiao S, Huang L, Yang B (2019). Genetic correlation of fatty acid composition with growth, carcass, fat deposition and meat quality traits based on GWAS data in six pig populations. Meat Science.

[ref-51] Zhao L, Li F, Liu T, Yuan L, Zhang X, Zhang D, Li X, Zhang Y, Zhao Y, Song Q, Wang J, Zhou B, Cheng J, Xu D, Li W, Lin C, Wang W (2022). Ovine ELOVL5 and FASN genes polymorphisms and their correlations with sheep tail fat deposition. Gene.

[ref-52] Zhou Y, Xu Z, Wang L, Ling D, Nong Q, Xie J, Zhu X, Shan T (2022). Cold exposure induces depot-specific alterations in fatty acid composition and transcriptional profile in adipose tissues of pigs. Frontiers in Endocrinology.

[ref-53] Zhou C, Zhang J, Ma J, Jiang A, Tang G, Mai M, Zhu L, Bai L, Li M, Li X (2013). Gene expression profiling reveals distinct features of various porcine adipose tissues. Lipids in Health and Disease.

