# Supplementary figures and images for: Differences in histomorphology and expression of key lipid regulated genes of four adipose tissues from Tibetan pigs
Source: PeerJ. 2023 Jan 9;11:e14556. doi: 10.7717/peerj.14556 (PMC9835692; doi:10.7717/peerj.14556)

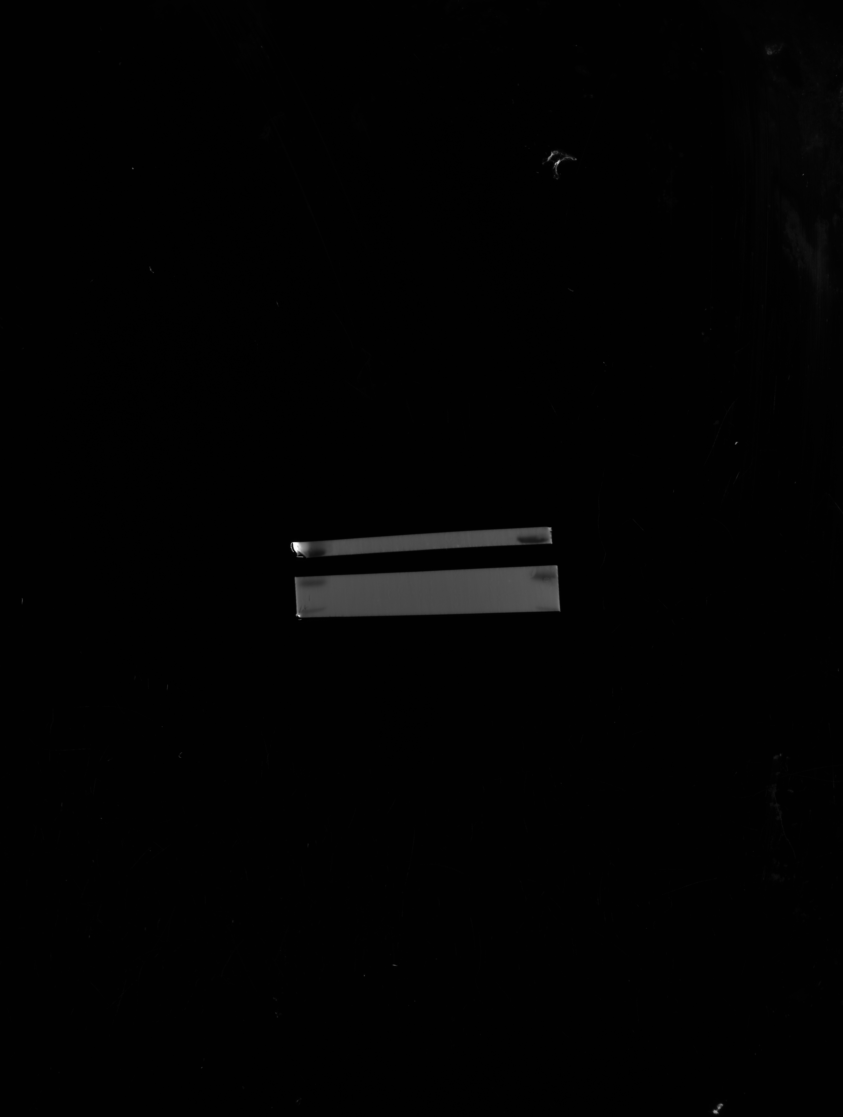

Supplement: Supplemental Information 2 [file peerj-11-14556-s002.zip › WB/FABP4-1-marker.tif]

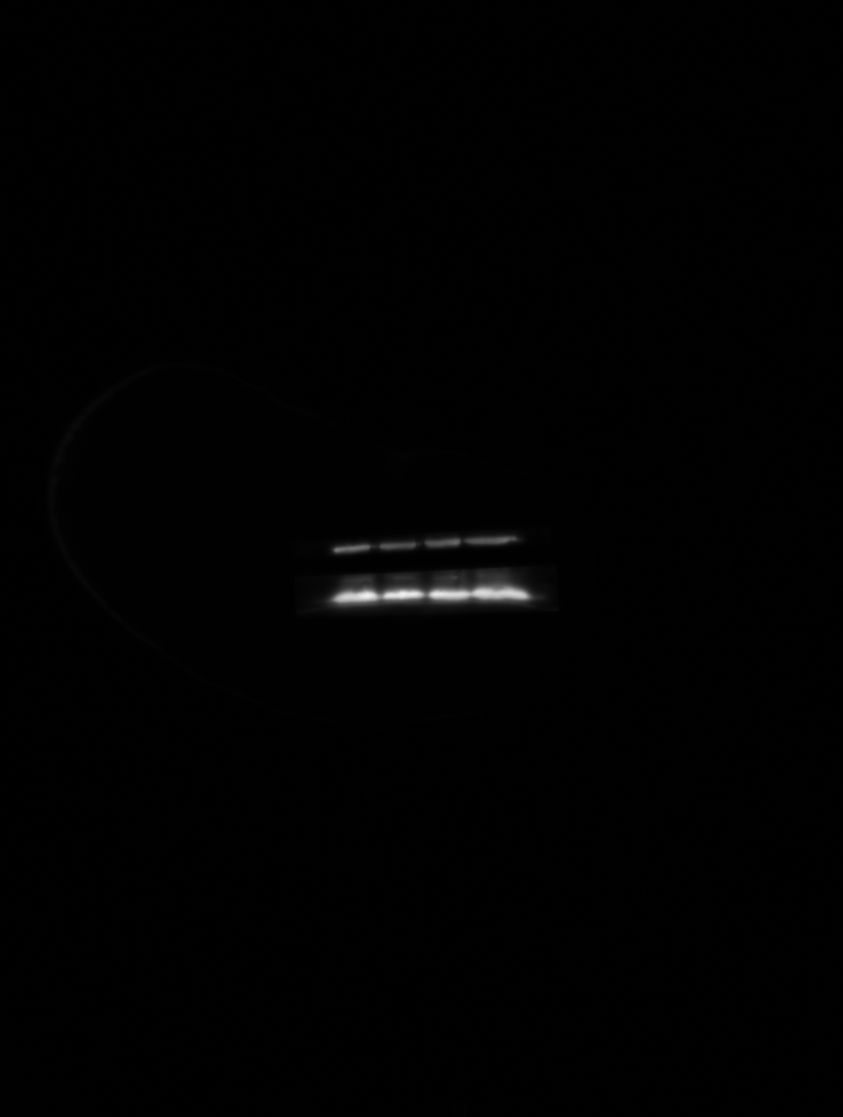

Supplement: Supplemental Information 2 [file peerj-11-14556-s002.zip › WB/FABP4-1.tif]

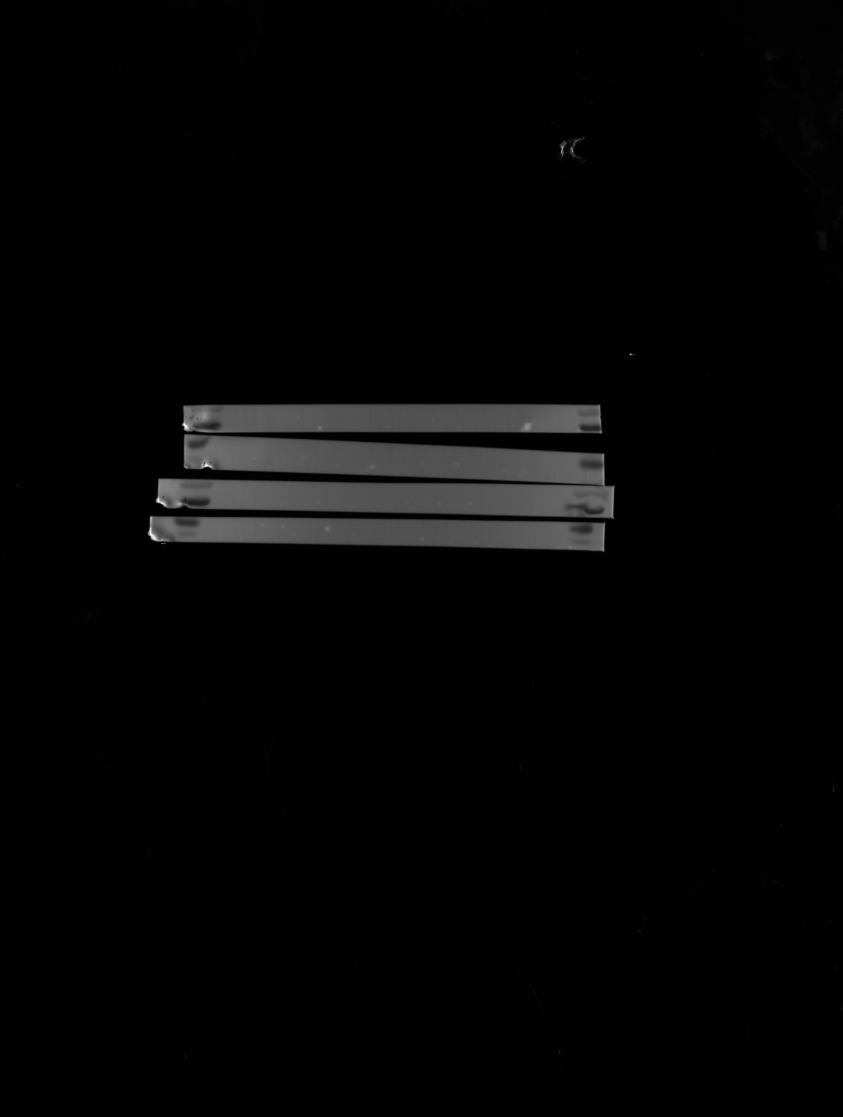

Supplement: Supplemental Information 2 [file peerj-11-14556-s002.zip › WB/FABP4-2-marker.tif]

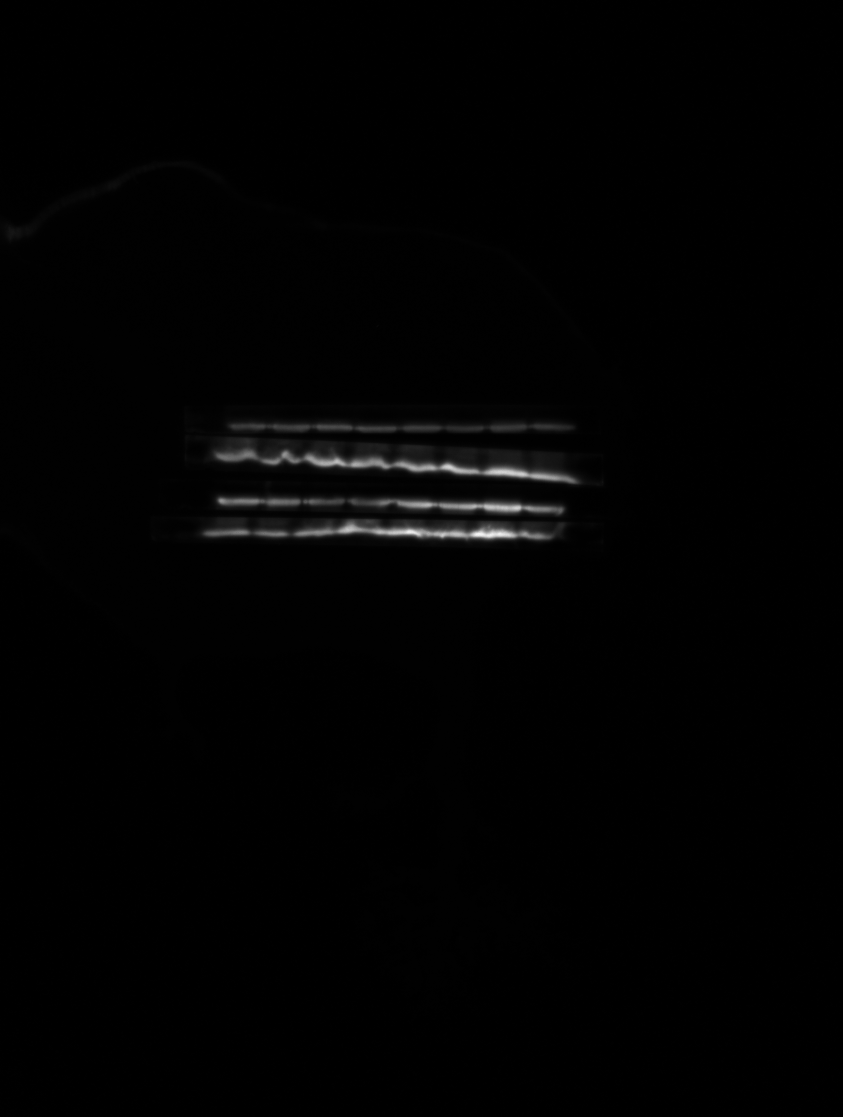

Supplement: Supplemental Information 2 [file peerj-11-14556-s002.zip › WB/FABP4-2.tif]

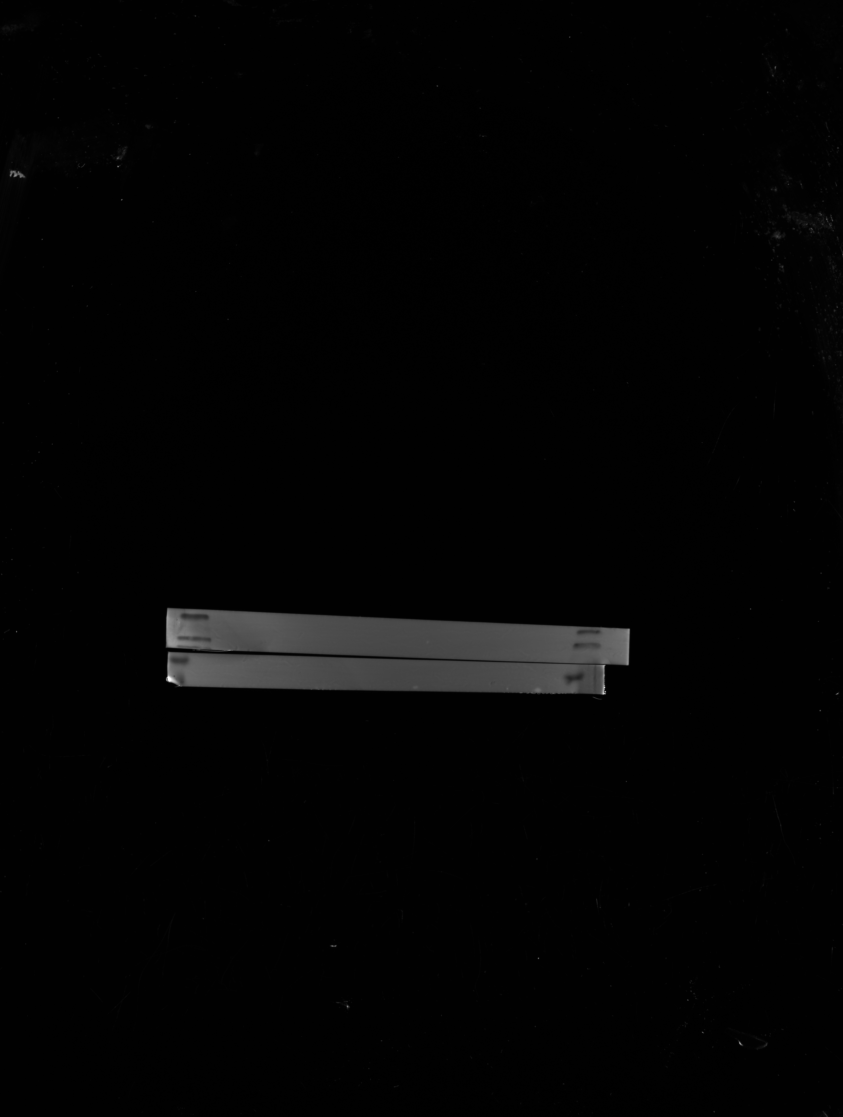

Supplement: Supplemental Information 2 [file peerj-11-14556-s002.zip › WB/FABP4-3-marker.tif]

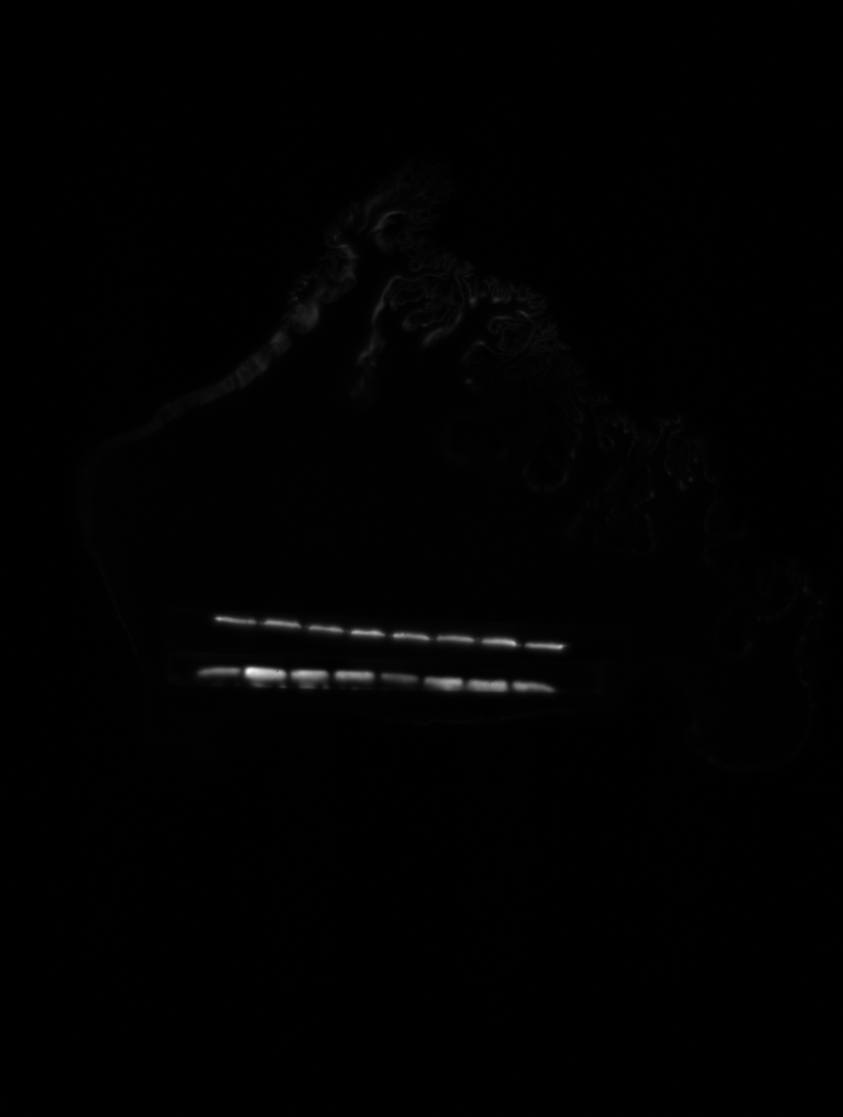

Supplement: Supplemental Information 2 [file peerj-11-14556-s002.zip › WB/FABP4-3.tif]

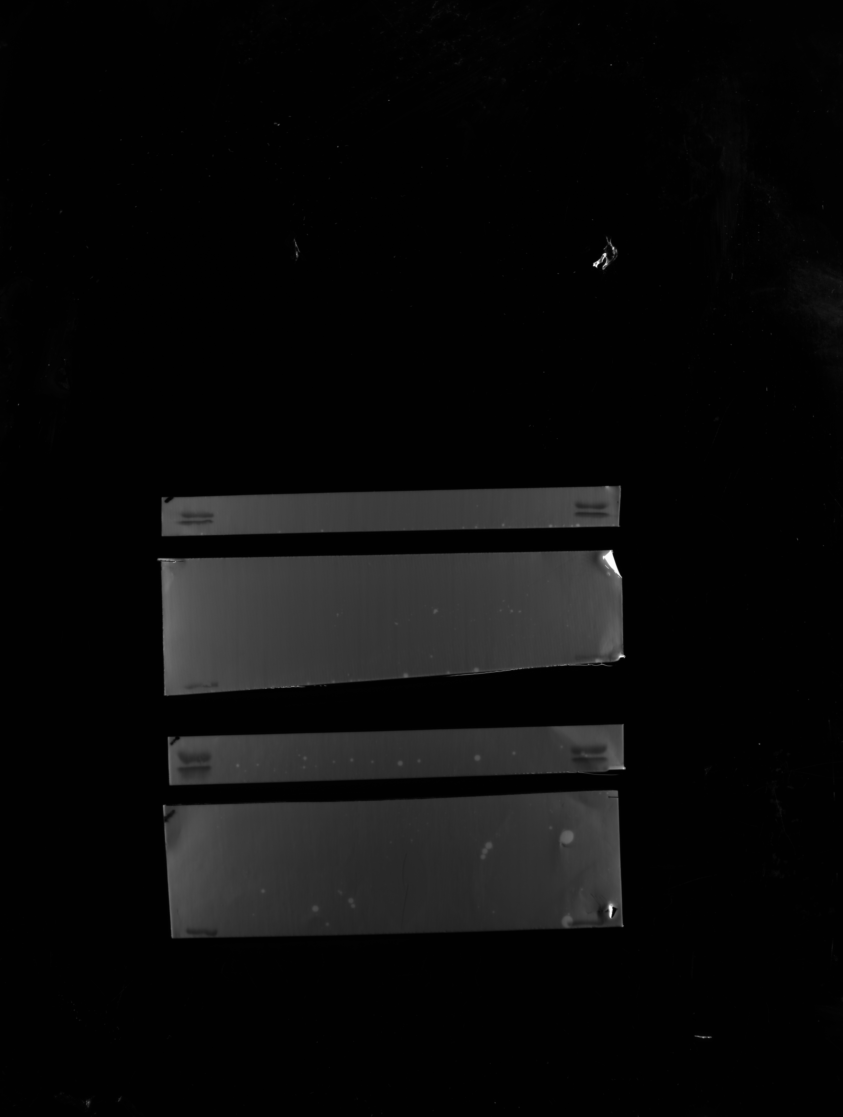

Supplement: Supplemental Information 2 [file peerj-11-14556-s002.zip › WB/FASN-1-marker.tif]

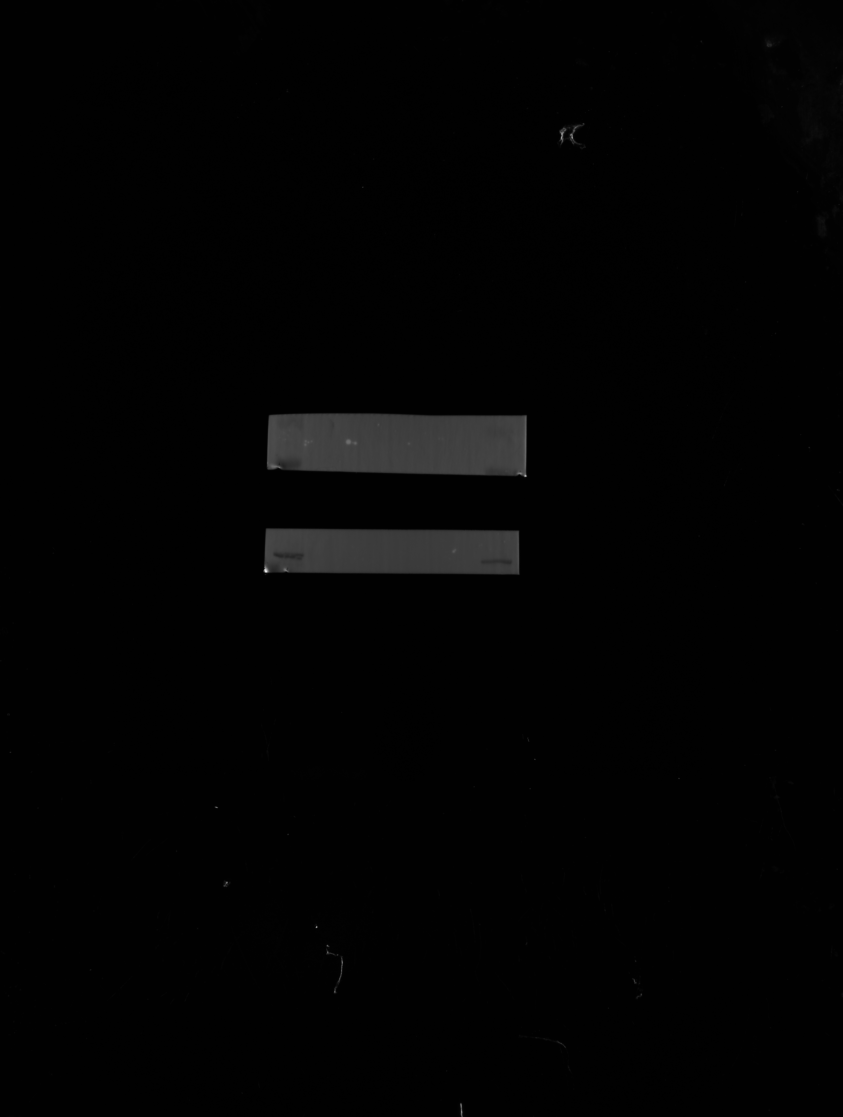

Supplement: Supplemental Information 2 [file peerj-11-14556-s002.zip › WB/FASN-2-marker.tif]

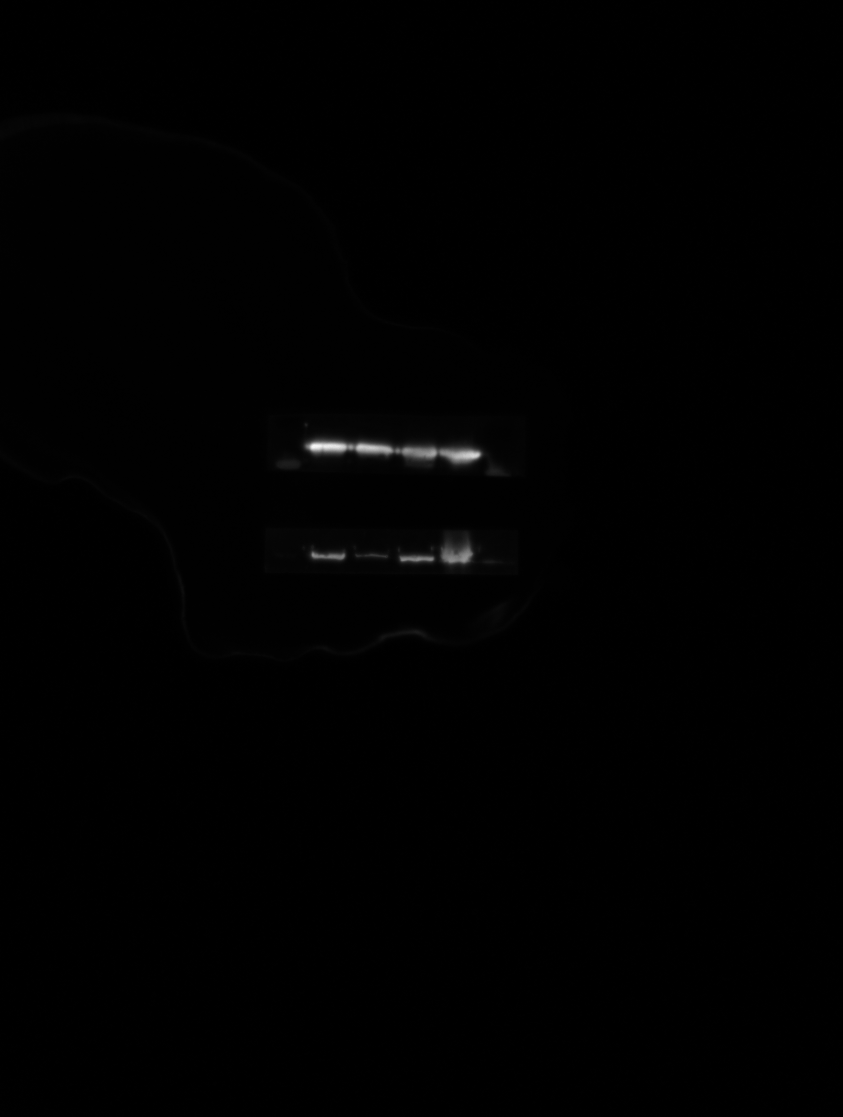

Supplement: Supplemental Information 2 [file peerj-11-14556-s002.zip › WB/FASN-2.tif]

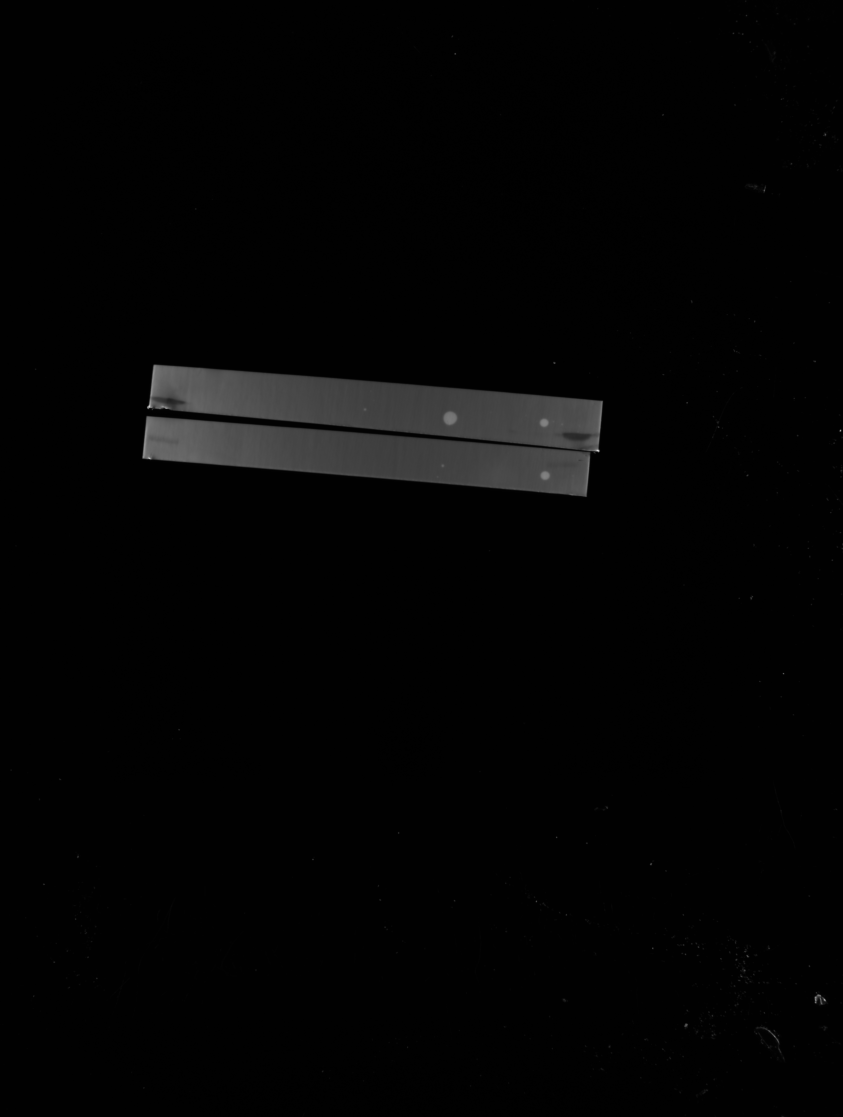

Supplement: Supplemental Information 2 [file peerj-11-14556-s002.zip › WB/FASN-3 -marker.tif]

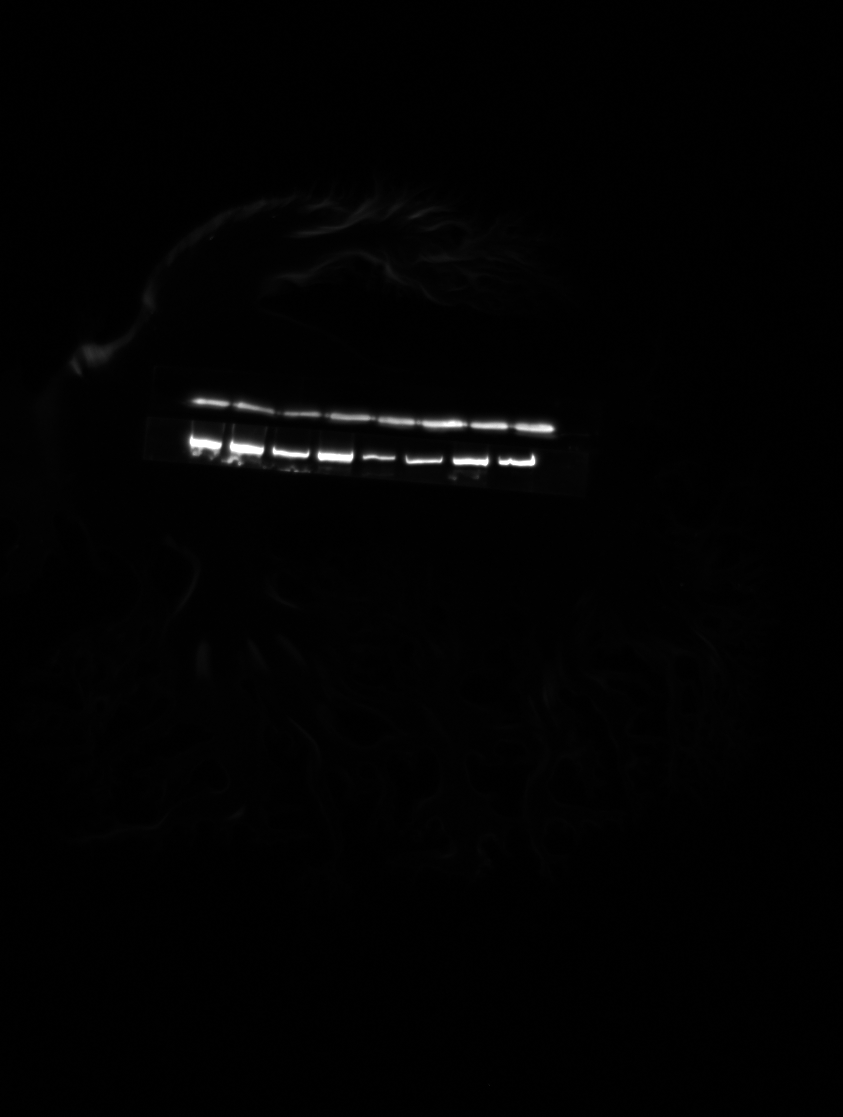

Supplement: Supplemental Information 2 [file peerj-11-14556-s002.zip › WB/FASN-3.tif]
